# SITC 2018 workshop report: *Immuno-Oncology Biomarkers: State of the Art*

**DOI:** 10.1186/s40425-018-0453-4

**Published:** 2018-12-04

**Authors:** Lisa H. Butterfield, Mary L. Disis, Bernard A. Fox, David R. Kaufman, Samir N. Khleif, Ena Wang, on behalf of the Society for Immunotherapy of Cancer Immuno-Oncology Biomarkers: State of the Art workshop speakers, Helen Chen, William D. Merritt, Sacha Gnjatic, Kara Davis, Ignacio Wistuba, James Lindsay, Stacey Adam, Fred Ramsdell, Kellie N. Smith, Davide Bedognetti, Chen Zhao, Evan Newell, Michael Angelo, David Furman, Marta Luksza, Pier Federico Gherardini, Priti Hegde, Martin Cheever, Steven Fling, Shawn Sweeney, Hongyue Dai, Catharine Young, John M. Kirkwood, Yana Najjar, Randy Sweis, Theresa LaVallee, Holden T. Maecker, Zoe Quandt

**Affiliations:** 1 Department of Medicine, Surgery and Immunology, UPMC Hillman Cancer Center, Pittsburgh, PA USA; 2 Cancer Vaccine Institute, University of Washington, Seattle, WA USA; 3 Earle A. Chiles Research Institute, Robert W. Franz Cancer Center, Providence Cancer Institute, Portland, OR USA; 4 Bill & Melinda Gates Medical Research Institute, Cambridge, MA USA; 5 Lombardi Comprehensive Cancer Center, Georgetown University School of Medicine, Washington, DC USA; 6 TICA Group, LLC, 9255 Towne Centre Dr, San Diego, CA 92121 USA

**Keywords:** Biomarkers, Cancer immunotherapy, PD-1/PD-L1, Checkpoint inhibitor

## Abstract

Identification of biomarkers in cancer immunotherapy that predict therapeutic response and/or limit adverse events are a critical need in the field. To address recent progress and hurdles around cancer biomarker development and utilization, the Society for Immunotherapy of Cancer (SITC) convened a workshop, “Immuno-Oncology Biomarkers: State of the Art,” on May 16–17, 2018. Topics discussed included challenges in handling biospecimens, identification and validation of new biomarkers, data sharing, and collaborating across disciplines to advance biomarker development. Panel discussions followed session presentations to help foster participant conversation and discuss future projects and collaborations. The results of the Workshop include the development of new initiatives for the SITC Biomarkers Committee.

## Introduction

Immunotherapy interventions, like those eliminating the interaction between the immune checkpoint proteins programmed death-1 (PD-1) and its ligand (PD-L1), have triggered responses in many types of cancer. Despite this exciting progress, only 20–30% of patients respond to immunotherapeutic interventions. The field needs to better understand treatment failure and how best to modify treatment for individual patients.

One strategy to increase patient response is predicated on the identification of reliable predictive biomarkers. Tumor PD-L1 expression may act as a predictive biomarker for cancer patients being treated with anti-PD-1/PD-L1 agents. Furthermore, the anti-PD-1 agent pembrolizumab was recently approved by the US Food and Drug Administration for the treatment of patients with solid tumors positive for the microsatellite instability (MSI-high) and/or DNA mismatch repair deficient (dMMR) biomarkers. Tumor mutational burden (TMB) has also become a biomarker of interest, with studies indicating differential predictive capabilities between TMB and PD-L1 status in patients being treated with anti-PD-1/PD-L1 agents [1].

There is an obvious urgent need to uncover more reliable biomarkers for predicting response to immunotherapy and guiding therapeutic decisions. With the advent of biomarkers into FDA-approved therapies and the further development of novel technologies including mass cytometry, gene expression profiling and whole exome sequencing, The Society for Immunotherapy of Cancer (SITC) hosted a two day program titled *Immuno-Oncology Biomarkers: State of the Art* on May 16–17, 2018, to bring together experts in the field of cancer immunotherapy to discuss opportunities, developments, and challenges in the cancer biomarker field (Table 1). In this report, we summarize the critical content of workshop presentations, ideas, and opinions from attendees (Table 2) and highlight next steps planned to facilitate cross-discipline collaboration to further advance cancer biomarker research and utilization.

**Table 1:** SITC 2018 Biomarkers Workshop Data Sharing Partnerships

Name of Institution	Objective (related to IO/Biomarkers)	Perspective
National Cancer Institute (NCI)	Address knowledge gaps in cancer immunotherapy to optimize clinical trial design. Developed the CIMAC-CIDC Network from the Cancer Moonshot Initiative to establish a standing network of laboratories and data commons for a systematic approach to biomarker discovery and validation.	Government
Cancer Immune Monitoring and Analysis Centers (CIMAC) and Cancer Immunologic Data Commons (CIDC) Network	Provide systematic support for correlative studies in immunotherapy trials through a standing network of laboratory centers (CIMACs) for immune profiling and analysis, and a data center (CIDC) for data repository, integration and analytical pipelines. The goal is to build the framework for a sustainable immuno-oncology data resource serving the NCI trial networks and funded programs and eventually the larger research community.	Government-Academic-Private Partnership
Foundation for the National Institutes of Health (FNIH)	Accelerate biomedical research through collaborations between NIH and leading public and private institutions. (E.g. FNIH manages the Biomarkers Consortium, a public-private biomedical research partnership for identification and development of high-impact biomarkers.)	Government
The Partnership for Accelerating Cancer Therapies (PACT)	Enhance ongoing efforts within the CIMAC-CIDC network to provide a systematic approach to IO biomarker investigation in clinical trials.	Public-Private Partnership
Parker Institute for Cancer Immunotherapy (PICI)	Utilize a collaboration-based model with specific considerations for standardized data and specimen sharing across all platforms.	Public-Private Partnership
Cancer Immunotherapy Trials Network (CITN)	Pair cutting edge clinical trials with correlative biomarker studies. Implementation of centralized operations, quality specimen collection and processing, competent biobanking, protocols and amendment management, real-time immune monitoring assays, collaborations with expert laboratories, and standardized data integration.	Academia
Biden Cancer Initiative	Develop and drive progress in cancer research, especially in assay/data standardization and harmonization - under Vice President Biden and Dr. Jill Biden’s Moonshot Initiative.	Non-profit
National Institute of Standards and Technology (NIST)	Apply transparent, open-sourced standards to cancer research and clinical care.	Public-Private Partnership
Alliance-NCI irAE Biorepository	Provide an efficient centralized repository for acquisition, organization and distribution of biospecimens in clinical trials; to improve treatment of severe irAEs.	Public-Private Partnership
Bill & Melinda Gates Medical Research Institute (The Gates Foundation)	Utilize IO biomarker strategies to develop drugs and vaccines for tuberculosis, malaria and enteric disease.	Non-profit

**Table 2:** Immune Profiling and Data Sharing Projects Presented at the SITC Workshop

Name of Project	Institution	Approach
Tumor Neoantigen Selection Alliance (TESLA)	Parker Institute for Cancer Immunotherapy	Support efforts to develop safe and effective neoantigen vaccines through effective neo-epitope prediction algorithms and high-quality epitope validation sets.
MANAFEST	Johns Hopkins Bloomberg-Kimmel Institute for Cancer Immunotherapy	An assay that combines whole exome sequencing, T cell receptor sequencing, and bioinformatics to identify targetable mutation-associated neoantigens.
APOLLO	MD Anderson Cancer Center	Biomarker discovery by analyzing tissue samples collected during pre-treatment, treatment and progression.
ECOG 1608 Biomarker project	Sidra Medicine Doha and ECOG-ACRIN	Combination of cellular immune monitoring assays, in vitro stimulation, protein profiling, genotyping, and transcriptomic analysis of PBMCs to estimate the proportion and functional orientation of immune cell subtypes.
Immunoscore/Immunoprofiling	Society for Immunotherapy of Cancer (SITC)	Objectively measure tumor immune infiltrates using digital imaging technology to demonstrate correlations with patient prognosis. Develop standards for next generation multiplex assays.
IBEX (Iterative Bleaching Extends Multi-pleXity)	National Institutes of Health	An iterative staining method detecting more than 40 protein markers to highlight immune-tumor interactions.
Peptide MHC tetramer staining	Agency for Science, Technology and Research	To identify antigen-specific T cells while preserving phenotypic profiles, and without requiring in vitro expansion.
Multiplexed ion beam imaging (MIBI)	Stanford University	Determine what cell phenotypes are present in a sample, how the discovered phenotypes are spatially distributed relative to one another, and how identified phenotypes are related to a disease state.
1000 Immunomes Project (1KIP)	Stanford University	A systems biology approach to the discovery of biomarkers associated with systemic chronic inflammation.
Neoantigen Fitness Model	Icahn School of Medicine	A mechanistic model representing the process of immune cell neoantigen recognition to predict tumor response to therapy.
imCORE global network	Genentech	Centralized testing of samples for data quality monitoring and public access to standardized clinical trial data.
Project GENIE (Genomics Evidence Neoplasia Information Exchange)	American Association for Cancer Research (AACR)	An international cancer registry that links clinical genotypes to patient outcomes.
Oncology Research Information Exchange Network (ORIEN)	M2Gen	A cancer center alliance based on the common use of the Total Cancer Care (TCC) protocol to accelerate cancer discovery through collaborative learning, partnership and data sharing.
Sparkathon Project TimIOs	Society for Immunotherapy of Cancer (SITC)	Cross-study analysis of patient response to help further the understanding of tumor heterogeneity in treating patients with immunotherapy.

## Main text

### Session I: State of the art - ongoing efforts in Cancer immune therapy

#### State of the field and collaborative efforts

Helen Chen, MD (National Cancer Institute, Rockville, Maryland, USA) introduced the workshop by discussing the importance of biomarkers through deep tumor and immune profiling in assessing how an agent or combination effects tumor cells, T cells, the tumor microenvironment, and cancer immunity and what determines response or resistance to a given therapy. Dr. Chen stressed that correlative studies involving biomarkers are necessary in order to address the knowledge gaps in cancer immunology and immunotherapy and to optimize clinical trial design. However, challenges include biological and technical complexity of tumor and immune profiling and the need to move from single trial to multi-trial analyses, which requires standardization in assays, data processing and analysis including normalization, scoring and reporting. Thus, the National Cancer Institute (NCI) developed the Cancer Immune Monitoring and Analysis Centers (CIMAC) and the Cancer Immunologic Data Commons (CIDC) Network from the Cancer Moonshot Initiative. The goal of these centers is to establish a pre-funded, standing network of laboratories and data commons for a systematic approach to biomarker discovery and validation, with the immediate goal of supporting the NCI-funded immunotherapy (IT) trials and the long-term goal of building the framework for a sustainable immuno-oncology (IO) data resource serving the larger research community.

William D. Merritt, PhD (National Cancer Institute, Rockville, Maryland, USA) discussed the goals and structure of the CIMAC-CIDC network. The primary goal of this network is to support individual trials and maximize translational potential by utilizing the collective power of correlative studies across the NCI trials networks and funded programs. This network of standing laboratories and database resources functions by coordinating activities for biomarker discovery and validation in order to develop molecular signatures that define immune response. Within the network there are four CIMACs (The University of Texas MD Anderson Cancer Center, Icahn School of Medicine at Mount Sinai, Dana-Farber Cancer Institute and Stanford University) and one CIDC (Dana-Farber Cancer Institute). CIMACs utilize multidisciplinary teams to perform assays and data analysis using biospecimens from NCI-funded IT trials. All CIMACs work as a network to collaborate on eligible trials, including early phase I and phase II immunotherapy trials, and are overseen by the Laboratory Coordinating Committee (LCC). As CIMACs accumulate biomarker data, the CIDC provides a data integration platform for both clinical and laboratory data to serve the network and the IO community.

#### Presentation by four individual CIMAC institutions and CIDC on opportunities and challenges of working in the network

Sacha Gnjatic, PhD (Icahn School of Medicine at Mount Sinai, New York, NY, USA) provided an overview of the assay platforms available across the CIMAC network. Dr. Gnjatic noted the importance of a multidisciplinary approach, utilizing the input from immunologists, pathologists, geneticists, tumor biologists, bioinformaticists and statisticians, in an ideal immune monitoring program balancing innovation with standardization, using analytically validated assays to minimize variability.

Immune response monitoring should occur both at the tumor site and at the tumor periphery. The tissue response is measured commonly by multiplex immunohistochemistry (IHC) or immunofluorescence, as well as immunogenomic approaches including whole exome sequencing (WES)/RNAseq, TCR sequencing, Nanostring/RT-PCR, and single cell sequencing. Immunopathology expertise is critical to discern immune cell type and abundance within the tumor microenvironment, while immunogenomics provide detailed characterization of both immune and tumor cells, including tumor mutational burden (TMB) to predict potentially targetable epitopes. Peripheral immune responses are measured for cellular composition and function by mass cytometry from blood, or for soluble analytes or antibody profiling from serum, as well as for microbiome composition from stool. Blood and tumor tissues are collected at multiple time points throughout immunotherapies and analyzed in a multiscale manner from organ imaging, to tissue mapping, to cellular mapping, down to molecular mapping. The overall data is then compiled and shared via the CIDC. The ultimate goal of the CIMAC laboratories is to define novel predictive biomarkers of cancer immunotherapies, by relying on harmonized technologies and protocols. To accomplish this, CIMACs are working to integrate datasets to define profiles across specific cancer types or interventions.

#### Clinical trial working groups within CIMAC-CIDC network

Kara Davis, D.O. (Stanford University, Stanford, California, USA) discussed how CIMACs can support IO clinical trials. Biomarker discovery during IO trials is critical to better understand patient therapeutic response. Dr. Davis referenced the phase I/II COG study, ADVL1412, assessing safety and efficacy of nivolumab as single-agent or in combination with ipilumumab in pediatric patients with solid tumors [2]. Challenges faced for optimal biomarker integration in this trial included cost, tissue availability, understanding optimal time points for tissue collection, competing interests (industry sponsor interests, regulation, investigator goals, patients’ needs), and logistics (collecting/shipping high quality samples for biobanking).

As such, the CIMAC/CIDC network is establishing a clinical trials working group to improve and standardize cutting-edge, correlative biological studies to be integrated into clinical trials. This working group will serve as a liaison between trial investigators and CIMACs to optimize trial design and utilization of CIMAC resources.

#### Challenges facing CIMACs in patient sample acquisition

Ignacio Wistuba, MD (The University of Texas M.D. Anderson Cancer Center, Houston, TX, USA) discussed challenges faced by CIMACs concerning processing and handling of patient samples and biobanking. One challenge is that tissue samples are inconsistently collected from patients enrolled in clinical trials. The importance and necessity to obtain fresh research samples for mechanistic studies and biomarker discovery are well appreciated but not universally implemented in clinical studies. Due to diverse sample collection methodologies, sample handling procedures, storage and shipping conditions, collected samples are not always viable or suitable for downstream immune, cellular and molecular assays. Another challenge is analysis of resected tumor tissue in the neoadjuvant setting. No consensus currently exists on when to collect tumor tissue and how to establish pathological response. One solution to these challenges is to incorporate pathologists in the design of the clinical trials and in sample collection, quality assessment, and tissue-based analyses. Overall, it is clear that integrated logistics in clinical research and sample processing are urgently needed in the immuno-oncology field.

#### Database and informatics issues experienced by CIMACs and the CIDC

James Lindsay, PhD (Dana-Farber Cancer Institute, Boston, MA, USA) discussed the workflow related to data analyses conducted throughout the cancer immune monitoring and analysis centers (CIMAC) and the cancer immunologic data commons (CIDC) network. Samples from eligible clinical trial cohorts will be sent to a CIMAC for molecular analyses where data is standardized and uploaded into a cloud based, central data repository. The CIDC is implementing standardized bioinformatics workflows and supporting integrative biomarker analyses by leveraging the cBioPortal for Cancer Genomics. The CIDC is piloting its platform in conjunction with a small number of trials selected by the network.

#### PACT: A public-private partnership to aid standardization of immune therapy biomarkers

Stacey Adam, PhD (Foundation for the National Institutes of Health (FNIH), Bethesda, MD, USA) presented on The Partnership for Accelerating Cancer Therapies (PACT). PACT is working to enhance ongoing efforts within the CIMAC-CIDC network by leveraging a 5-year, $220 million precompetitive public-private research collaboration - managed by the FNIH - for the standardization of biomarker and related clinical data to support the selection and testing of promising therapeutic combinations. PACT was formed under the Cancer Moonshot and is made up of the NIH/NCI, FDA and 12 global industry partners. PACT aims to provide a systematic approach to immune and related oncology biomarker investigation in clinical trials by supporting development of standardized biomarkers and assays to advance biomedical research and training collaborations among government, universities, industry and no-for-profit organizations. The PACT Trial Selection Working Group will select eligible trials in a flexible manner, allowing for multiple IO trial types to be considered for funding.

#### Neoantigen selection and the TESLA program

Fred Ramsdell, PhD (Parker Institute for Cancer Immunotherapy, San Francisco, CA, USA) described the Tumor Neoantigen Selection Alliance (TESLA). TESLA was created by the Parker Institute for Cancer Immunotherapy and the Cancer Research Institute (CRI) in the fall of 2016. A bioinformatics collaboration, TESLA is comprised of a global alliance which includes scientists from over 35 of the world’s leading neoantigen research groups in academia, industry and nonprofit. The goal of this consortium is to support efforts to develop safe and effective neoantigen vaccines by delineating the key parameters necessary for effective neo-epitope prediction algorithms and generating high-quality epitope validation sets. The necessity of TESLA is exemplified in the fact that there are currently 675 active cancer vaccines, another 372 in clinical development, and yet nothing has proven particularly effective in large scale trials.

#### The MANAFEST assay for monitoring anti-tumor immunity

Kellie N. Smith, PhD (Johns Hopkins Bloomberg-Kimmel Institute for Cancer Immunotherapy, Baltimore, MD, USA) presented on The Mutation-Associated Neoantigen Functional Expansion of Specific T Cells (MANAFEST) Assay – a technology that combines whole exome sequencing, T cell receptor (TCR) sequencing, peptide-stimulated cultures and bioinformatics to identify targetable mutation-associated neoantigens (MANAs) found in a tumor. Ultimately designed to detect a patient’s immune response to their own tumor, MANAFEST explicitly measures antigen-specific TCR clonotypic amplifications following patient T cell in vitro stimulation with identified peptide neoantigens [3]. MANAFEST can be used to detect patient response to checkpoint blockade and may be particularly useful in patients with tumors that have low mutational burden. This assay overcomes common problems with the comparable ELISpot assay that may underestimate the anti-tumor immune response. Translationally, utilization of this assay could potentially lead to correlation of antigen-specific T cell dynamics with clinical parameters such as radiographic response, as well as potentially identifying and measuring dynamics of antigen-specific clones throughout treatment or disease progression [3].

#### Comprehensive biomarker discovery in immunotherapy clinical trials: A longitudinal approach

Dr. Wistuba concluded Session I by highlighting MD Anderson Cancer Center’s Moonshot Initiative program APOLLO (Adaptive Patient-Oriented Longitudinal Learning and Optimization), which is working to collect and process tissue, blood, and other fluids for clinical trials, including immunotherapy-related trials. As a cross-disciplinary collaboration between MD Anderson’s analytical labs, bioinformatics systems, clinical teams and research activities, APOLLO aims to discover biomarkers by analyzing tissue samples collected pre-treatment, during treatment and throughout progression. For each participating patient, five core biopsies from solid tumor or bone marrow aspirates from blood cancers will be analyzed at each time point. High quality specimens and longitudinal approach are the key aspects of this program. A pilot APOLLO melanoma trial involving tumor tissue analyses of 54 patients being treated with anti-CTLA-4 blockade validated this strategy [4]. In the future, it is intended that researchers can use data generated from the APOLLO program to identify biomarkers associated with immune responsiveness and toxicity.

Davide Bedognetti, MD, PhD (Sidra Medicine Doha, Qatar) concluded the session with preliminary data on the ECOG 1608 Biomarker project (a collaboration between Sidra, the University of Pittsburgh (Dr. Butterfield Lab) and ECOG-ACRIN)). This trial (P.I. Stephen Hodi) tested ipilimumab +/− GM-CSF in melanoma [5]. The biomarker discovery under this project combines cellular immune monitoring assays (flow cytometry), in vitro stimulation (antigen-specific cytokine production), protein profiling (Luminex), genotyping (Illumina MEGAEX Array), and transcriptomic analysis (Illumina RNA-seq) of peripheral blood mononuclear cells (PBMC). Thus, by using deconvolution approaches to estimate the proportion and functional orientation of immune cell subtypes, combined with a modular framework for subsequent analysis of any PBMC dataset, [6] shared and treatment-specific transcriptomic perturbations were detected and correlated with outcome.

#### Session I panel discussion outcome

The panel discussed issues of standardization of assays through the CIMAC network and the need for assay development to keep pace with research in the field, the need of implement data sharing to facilitate integrative analysis, the advantages of imaging whole slides versus regions of interest, variation between analytical platforms, inadequate and non-standardized testing of tissue samples by pathologists outside of academic hospitals, complexities faced in deep learning models, and biomarkers for tumor reactivity compared to immune toxicity.

### Session II: Identification, analysis and validation of biomarkers

#### Immunoscore task force: A SITC-led global study

Bernard Fox, PhD (Earle A. Chiles Research Institute, Robert W. Franz Cancer Center, Portland, Oregon, USA) introduced the SITC-led Immunoscore Task Force to attendees. The Immunoscore Validation Project is a global collaboration, including over 14 countries, with the purpose of introducing immune parameters into tumor classification systems. The immunoscore itself is an objective measurement of tumor immune infiltrates using digital imaging technology [7]. Using standardized procedures, the densities of CD3+ and cytotoxic CD8+ effector T cells in both the tumor and invasive margin of each patient’s cancer were processed for immunohistochemistry. Tumor infiltrating T cell counts were then quantified and compared by digital pathology. An Immunoscore for each patient was derived from the mean of four percentiles based on cell densities [8]. Positive correlations between tumor immune infiltrates and prognosis of patients with melanoma [9], head and neck cancer [10], breast cancer [11], ovarian cancer [12], and colorectal cancer [13] had been previously reported, but never validated. Thus, SITC provided logistical and infrastructure support to analyze the Immunoscore of colon cancer samples collected from patients worldwide. Analyses revealed that the colon cancer patients with a low Immunoscore experienced faster progression than those with a high Immunoscore. Furthermore, multivariate analyses showed that Immunoscore was the most significant prognostic biomarker for time to recurrence (TTR), disease free survival (DFS) and overall survival (OS). Interestingly, the prognostic outcome based on a patient’s Immunoscore was independent of the patient’s MSI status. As such, Immunoscore may serve as the basis for a standardized immune-based assay for the classification of cancer allowing for stratification of patients enrolled in clinical trials.

#### Multi-parametric biomarkers I: Seeing is believing – Dissecting local immune-tumor interactions with advanced imaging techniques

Dr. Chen Zhao (National Institutes of Health, Bethesda, MD, USA) presented the recent achievements in advanced imaging techniques at Dr. Ronald N. Germain’s lab and how to use these tools to dissect the local immune-tumor interactions. They have successfully applied histo-cytometry, an innovative and powerful imaging analysis tool developed at Dr. Germain’s lab earlier, to examine the cell signaling status and function along with phenotype and spatial information in the tumor microenvironment of genetically engineered mouse models and patient samples [14]. To achieve higher complexity, they developed IBEX (Iterative Bleaching Extends Multi-pleXity), which is an iterative staining method detecting more than 40 protein markers. Besides that, they are moving forward by combining iterative staining with iterative RNA FISH using Ce3D (clearing-enhanced 3D microscopy) to develop highly multiplex 3D datasets. This will help researchers to understand the complex tumor microenvironment and illuminate immune-tumor interactions.

#### Multi-parametric biomarkers II: High-dimensional profiling of cancer-specific vs. bystander tumor infiltrating T cells

Evan Newell, PhD (Fred Hutchinson Cancer Research Center, Seattle, WA, USA) presented on the role of T cells in cancer progression. There is a need to profile T cell phenotypes and antigen specificity to elucidate relevant populations in eliciting immune responses, ultimately assisting in the development of methods to be used in personalized medicine. High-dimensional T cell phenotypic profiling reveals a wide range of possible T cell phenotypes, especially within tumors. By profiling antigen-specific T cells, researchers can better understand what these profiles mean [15]. For this purpose, Newell’s group is using peptide MHC tetramer staining to identify antigen-specific T cells. In conjunction with mass cytometry, MHC tetramer staining provides a workflow for T cells to be probed with high levels of detail. Such methods allows for analysis of numerous candidate antigens while preserving phenotypic profiles and without requiring in vitro expansion. Specifically, this protocol simultaneously evaluates surface marker expression, functional capacity and antigen-specificity [16]. To allow for enhanced antigen-specificity and assessment of hundreds of different antigens in a single sample, analysis of this process is executed concurrently with a highly multiplexed method based on combinatorial coding of peptide-MHC tetramers followed by computational data analysis approaches to verify specificity of staining and determine the extent of cell diversity [17]. Long-term goals for development and application of this high-dimensional antigen-specific T cell profiling technology include finding novel therapeutic targets, discovery of more accurate biomarkers of clinical outcomes and prediction of immune response to IO therapies [18].

#### Multispectral imaging: Higher dimensionality imaging approach

Michael Angelo, MD, PhD (Stanford University, Stanford, CA, USA) presented on the multiplexed ion beam imaging (MIBI) working principles and how his team is using the MIBI workflow to explore the role of infiltrating immune cells in triple negative breast cancer [19]. MIBI is beneficial to researchers as it allows for staining of tissue samples with all mass labeled antibodies at once instead of using the standard cyclical staining process, as well as allowing for a stationary sample to be raster scanned with a particle beam in place of scanning the sample using lasers on a moving stage. Additionally, MIBI permits scanning at low resolution to scan the entire section with all antibody markers, identification of regions of interest (ROI) and the ability to rescan ROIs at higher resolutions. This technology is distinct from laser ablation inductively coupled plasma mass spectrometry (LA-ICPMS) used by CyTOF instrumentation. MIBI permits quantitative, multiplexed imaging of up to 100-metal isotope reporters directly in tissue at resolutions down to 200 nm, comparable to brightfield microscopy. Relative to imaging mass cytometers, MIBI offers an order of magnitude higher sample throughput and sensitivity while achieving up to five-fold higher imaging resolution. Such technological advancements have the potential to improve understanding of tissue composition through high-dimensional, quantitative analyses in situ [20, 21]. The goal of MIBI is to determine what cell phenotypes are present, how the discovered phenotypes are spatially distributed relative to one another, and how identified phenotypes are related to a disease state. Furthermore, for analysis of captured images, cells are segmented into single cell networks, clustered into groups, and then analyzed to compare composition across patient samples. Ultimately, the goal is to determine which cell types co-localize in a frequent and meaningful manner such that the compartmentalized phenotype correlates significantly with pathogenesis or clinical endpoints.

#### Machine learning I

David Furman, PhD (Stanford University, Stanford, CA, USA) presented the topic of Machine Learning as it is applied to multiscale immune monitoring in systemic chronic inflammation (SCI) and cancer. It is well established that chronic inflammation induces epigenetic modifications in key regulatory genes, which can lead to the initiation of cancer. Accumulating evidence have also demonstrated that chronic inflammation can lead to impaired immune system function leading to poor protection against pathogens and tumors, known as immunosenescence. However, there is no consensus regarding which biological markers define chronic inflammation and how they work to dampen the immune response. In order to advance clinical understanding of chronic inflammation, the 1000 Immunomes Project (1KIP) was started to provide a systems biology approach to the discovery of biomarkers associated with SCI. One thousand individuals were recruited at Stanford during the years 2008–2016. These ambulatory subjects were monitored for blood proteomics, cell frequencies, gene expression, in vitro responses to cell stimulations, HLA deep genotyping, and their serological responses to the influenza vaccine (in 600 of the individuals). In addition, clinical data was recorded to derive ‘immune metrics’ for health and disease. Deep learning algorithms were used to construct a simplified “inflammatory score” (immScore), which was used to create the first reference values for standardizing SCI.

#### Machine learning II: Predicting tumor evolution from immune interactions

Marta Luksza, PhD (Icahn School of Medicine at Mount Sinai, New York, NY, USA) discussed a computational approach to predict tumor evolution from immune interactions. This approach is based on a mechanistic model representing the process of neoantigen recognition by immune cells [22]. Her studies show that immune interactions involving neoantigen fitness (the likelihood of presentation by the major histocompatibility complex) and subsequent neoantigen quality (the ability of neoantigens to recognize T cells) can influence a patient’s response to immune checkpoint inhibitors [22]. As such, Dr. Luksza demonstrates a mathematical fitness model that can predict how tumors respond to immunotherapy by identifying the relative levels of neoantigen quality and fitness.

The goal of machine learning is to quantify the immunogenicity of neoantigens in an evolutionary model to predict tumor response to therapy. Dr. Luksza explains that immune interactions with tumor neoantigens affect a cell’s fitness such that a cancer cell recognized by T cells will not replicate as well as one that isn’t. Dr. Luksza presented the Neoantigen Fitness Model which was created to score the recognition potential of a peptide (s) by accounting for both components necessary for immune recognition: peptide’s presentation by MHC and subsequent recognition by T-cell receptors. Both components are quantified based on modelling the probability of the underlying biophysical interactions, namely of binding between a peptide and the MHC, and binding of a presented peptide and a host’s T-cell receptor.

The goal of using prediction models is to be able to rationalize health care decisions and understand or modify treatments. However, the high dimensionality of genomic sequences and genetic heterogeneity of tumors makes response prediction difficult as a single tumor may have multiple immune interactions due to its multiple neoantigens. This approach, which utilizes both a mechanistic model and biophysical immune interactions, employs prior knowledge regarding evolutionary dynamics to reduce the complexity of machine learning.

#### Session II panel discussion outcome

The Discussion panel recommended the need for reference materials for standardization, the need to move the Immunoscore towards a reimbursable pathology test, and the hurdle of tumor heterogeneity and limited biopsy samples.

### Session III: Data and specimen sharing

#### Cancer immunologic data commons (CIDC)

James Lindsay, PhD (Dana-Farber Cancer Institute, Boston, MA, USA) presented the CIDC’s view on data. The guiding principles behind the CIDC data sharing network include: 1. providing data quickly to researchers, 2. forming a standardized network of software and bioinformatics tools, and 3. focusing on software innovation unique to the biomarker space. Additionally, all content needs to meet security requirements and be standardized prior to access as described by the FAIR (Findable Accessible Interoperable Reusable) data ethos, emphasizing that data harmonization is imperative for effective data sharing.

#### Dating sharing for PACT and FNIH

Stacey Adam, PhD (Foundation for the National Institutes of Health, Bethesda, MD, USA) spoke on key data sharing principles used within ongoing FNIH public-private partnerships (PPP). One discussed ‘key’ for current and future PPPs is flexibility to allow the needs of the partnership dictate the model. This includes considerations such as the motivating scientific need for involved stakeholders, sharing models, availability for broad public research use, inclusivity of appropriate partners, and IP policy considerations.

The Partnership for Accelerating Cancer Therapies (PACT) is one of the newer FNIH PPPs. PACT-selected trials can be conducted by a number of sources, but a set of standardized biomarkers will be run by the CIMACs. The trials receive supplemental funding in exchange for depositing their sponsored biomarker and accompanying de-identified clinical data to the CIDC for public use. Logistically, CIMACs deposit raw data from their involved trials into the CIDC, where data is de-identified and analyzed for publication and IO community use. PACT utilizes a tiered data access structure, providing data access first to trial investigators and sponsors, next to PACT partners, and then the public. Despite progress, several challenges remain including building data sharing infrastructure, trial recruitment speed, patient consent, potential international regulatory approval, standardization to a common clinical data standard (CDISC), and IP guidelines.

#### Ongoing advanced correlative assays at the Parker Institute for Cancer immunotherapy (PICI)

Pier Federico Gherardini, PhD (Parker Institute for Cancer Immunotherapy, San Francisco, CA, USA) discussed efforts at PICI to deal with challenges from advanced correlative assays, from both a logistical and data analysis perspective. PICI utilizes a collaboration-based model for their ongoing projects, with specific considerations for data and specimen sharing across all platforms. On the logistics side, planning in advance for appropriate sample collection and aliquoting is fundamental if several assays are to be run in parallel. Moreover Dr. Gherardini underscored the challenges of standardization, even for well-established assays. On the data analysis side Dr. Gherardini highlighted how issues such as missing data, and small datasets make the application of Machine Learning challenging. To address these analytical challenges, PICI has assembled a central computational team that is well versed in biology and works in close collaboration with the investigators. The underlying assumption is that deeper molecular characterization of the samples will lead to more opportunities for novel discovery.

#### Industry perspective

Priti Hegde, PhD (Genentech, San Francisco, CA, USA) discussed an industrial perspective on data and specimen sharing. Driven by the belief that high-quality data is critical in advancing progress against cancer, industry is an advocate of centralized sample testing and data quality monitoring as well as fostering public access to standardized clinical trial data. There is a need for clear hypotheses and study design regarding statistical significance in biomarker analyses, viable specimen acquisition protocols, and assay qualification. To implement centralized testing of samples for data quality monitoring and public access to standardized clinical trial data, Dr. Hegde’s institution initiated imCORE, a global research network of 21 academic centers, where scientists can readily share technology, data, and expertise. This is exemplified in a 326 patient Phase II urothelial bladder cancer clinical trial for atezolizumab, IMvigor210, where all RNAseq, WES, CD8 IHC, PD-L1, and outcome data has been made publicly available [23].

#### Academic perspective

Samir N. Khleif, MD (Georgetown University, Washington D.C.) shared an academic perspective on challenges and opportunities encountered in data sharing. It has become clear that data sharing enables the exploration of topics and correlations potentially disregarded by initial analyses. However, although data sharing is vital to the development of clinical trials to reduce both time and cost, academics face many challenges.

Academics often face challenges in accepting the validity of data from other sources. Ethical considerations also must be accounted for, including responsible data management, verification, and ownership. Legal challenges including privacy regulations and data exchange between countries can also become problematic. Cultural challenges can arise as well, including the reluctance to share data in academia, claiming credit, as well as promotion and tenure procedures. IP ownership and high costs of implementing data sharing solutions in addition to technical challenges such as data storage, management, standardization, and harmonization can also serve as barriers.

#### Cancer immunotherapy trials network (CITN): Data management and specimen sharing

Martin Cheever, M.D., and Steven Fling, PhD (Fred Hutchinson Cancer Research Center, Seattle, WA, USA) presented on the Cancer Immunotherapy Trials Network (CITN). Funded by the National Cancer Institute and the Fred Hutchinson Cancer Research Center, the CITN is comprised of 30 member sites (29 US-based, 1 Canadian), and is working to accelerate immunotherapy development by pairing cutting edge clinical trials with correlative biomarker studies. Thus far, anti-PD1 and anti-PD-L1 immunotherapy dominates the field, making it likely that many patients will be considered for anti-PD1/PD-L1 therapy alone or in combination. Patients failing these treatments will become the largest population of cancer patients. With the many available agents to be potentially used in combination with checkpoint inhibitors, rapid biomarker analyses are imperative to discern optimal agents with which to move forward. To address this challenge, the CITN implemented a comprehensive framework, including centralized operations, quality specimen collection and processing, competent biobanking, management to match protocols and amendments, real-time immune monitoring assays, close collaborations with expert laboratories, and a standardized procedure for data integration. This framework can help researchers rapidly initiate trials and better understand treatment failure.

The CITN has put into place two integrated repositories: BioSpecimens (BSI-II) and Correlative Science Data (LabKey). BSI is an interface used by CITN to communicate with clinical sites by posting information in real-time and tracking all specimens. Labkey is a web-based system for managing data, allowing users to visualize study organization. Any user with access can view raw and analyzed data within and across studies. Alignment of these two systems and coordination of data between them is fundamental in accelerating progress.

#### AACR project GENIE

Shawn Sweeney, PhD (American Association for Cancer Research (AACR), Philadelphia, PA, USA) presented on AACR’s Project GENIE (Genomics Evidence Neoplasia Information Exchange), an international cancer registry that links clinical genotypes to patient outcomes with the goals of driving clinical decision making and translational research. The GENIE registry currently contains data from over 48,000 sequenced tumors from the eight founding institutions and has recently completed an expansion to a total of19 participating institutions and their associated cancer centers. Of the participants, there are 14 medical centers from the United States, 4 from Europe and 1 Canadian institution.

Project GENIE collects both genomic and clinical data, each with distinct analytical pipelines and processes. Concerning genomic data, Project GENIE analyzes several biomarkers, including microsatellite instability (MSI), DNA mismatch repair deficiency (dMMR), tumor mutational burden (TMB), and holds the ability to analyze other co-occurring mutations. Currently, a pragmatic set of baseline clinical data are collected on every patient with more detailed clinical data and outcomes collected on specific cohorts. The consortium is currently in the process of expanding the data collected as part of the baseline through a multiyear staged project. In addition to the genomic and clinical data, the BAM files; nucleic acid libraries; stained slides; and in many cases, tissue, are available and can be used to drive further discovery. One ongoing project is working to correlate calculated MSI, TMB, and dMMR results with SOC testing (PCR and IHC) as well as outcomes to immune checkpoint blockade therapy.

#### Oncology research information exchange network (ORIEN)

Hongyue Dai, PhD (M2Gen, Tampa, FL, USA) presented on the Oncology Research Information Exchange Network (ORIEN) - a cancer center alliance of 18 US-based academic medical centers grounded on the common use of the Total Cancer Care (TCC) protocol. The mission of ORIEN is to accelerate cancer discovery through collaborative learning, partnership and data sharing. TCC, which was developed at Moffitt Cancer Center, is the largest prospective observational study of its kind in the cancer arena, allowing for patients to be followed throughout their lifetime to better identify, meet, and ultimately predict clinical need. ORIEN Avatar – an ORIEN project - focuses on cohorts of high-risk patients and those with unmet medical needs, including clinical trials. The project collects detailed longitudinal clinical data, and generates uniform in-depth molecular data, to promote and enable both the matching of patients to clinical trials as well as collaborative research among cancer centers and between cancer centers and industry partners.

#### Biden Cancer initiative

Catharine Young, PhD (Biden Cancer Initiative, Washington, D.C.) next presented the Biden Cancer Initiative: Ending cancer as we know it. The Biden Cancer Initiative brings to life Vice President Biden’s and Dr. Jill Biden’s vision of a day when we can effectively diagnose, treat and care for every patient afflicted by cancer. The Biden Cancer Initiative recognizes that clinical analyses cannot be compared across institutions, severely limiting second opinions, collaboration, and access to medical records, thus placing a significant need for assay standardization and harmonization. As such, the Biden Cancer Initiative is currently working on a pilot study testing an arrangement where patients, treatment centers and industry can contribute de-identified data for qualified visualization and analysis. In regard to standardization, the Biden Cancer Initiative is working with the National Institute of Standards and Technology (NIST) to convene public and private sector experts with the mission of bringing pre-existing, open-source standards to cancer research and clinical care. The core mission of this initiative is to create the cancer research and health care system that patients expect and think we already have.

#### Immune-related adverse events

John M. Kirkwood, MD (University of Pittsburgh and UPMC Hillman Cancer Center, Pittsburgh, PA, USA) next presented on inflammatory and autoimmune toxicities associated with immuno-oncology therapies. With the recent advancements and FDA approvals in immunotherapy, IO agents will be used in millions of patients with advanced disease, adding years of subsequent survival. With the most recent approvals of anti-PD1 agents for the adjuvant setting, even larger populations of patients who do not necessarily have late stage disease, and who are destined for relapse or progression will be treated. Toxicities experienced due to treatment with immunotherapies, or irAEs, pose a significant issue regarding future treatments and combinations for patients who may otherwise experience significantly improved long-term survival.

As such, there is a need to establish an efficient centralized repository for acquisition, organization and distribution of well-annotated biospecimens for translational studies to improve the understanding of molecular pathogenesis and treatment of severe irAEs. This need has been addressed through the Alliance-NCI irAE Biorepository, an intergroup consortium which aims to 1. Develop biospecimen and clinical data collections from retrospectively identified trial patients with irAEs; 2. Develop a mechanism for the prospective collection of data and biospecimens from trial patients with new onset irAEs to be analyzed using the irAE biorepository protocol; and 3. Pilot a prospective registry and biobanking trial for patients who develop irAEs receiving standard of care IO therapies at Washington University and Dana-Farber Cancer Institute. Better understanding of the mechanism (s) of IO induced autoimmune toxicities may aid in mitigation of these toxicities in the future.

#### Sparkathon project TimIOs

Yana Najjar, MD (University of Pittsburgh, Pittsburgh, PA, USA) and Randy Sweis, MD (University of Chicago, Chicago, IL, USA) discussed the TimIOs project, developed through SITC’s Sparkathon initiative, to conduct a pooled analysis of durable versus transient responders enrolled in immunotherapy clinical trials. SITC’s Sparkathon program brings together early-career scientists of different backgrounds to address current hurdles in the field of IO. The SITC Sparkathon Class of 2017, which created Team TimIOS, included 29 emerging leaders from global academic medical centers, US-based government agencies and one US-based private institution. TimIOs was granted funding to develop tools for advancing understanding of tumor heterogeneity and clinical response, and subsequently proposed a unified public-private consortium where TimIOs would act as an honest broker to facilitate cross-institution collaboration. TimIOs aims to build a platform that will help identify fundamental differences between two patient response cohorts: durable (partial or complete response longer than 2 years) vs transient (partial or complete response shorter than 6 months) responders, and elite responders (patients with complete response) vs rapid progressors.

#### Session III panel discussion outcome

The panel discussed identifying immune health through the use of standardized CyTOF panels within the CIMACs, analyzing CMV responses in cancer patients, examining immune health across diverse diseases, profiling immune competency and irAEs, whether immune toxicity associated with checkpoint blockade is related to the administration of the antibodies themselves or T cells, funding for development of therapeutic antibodies versus T cell research, how best to combine datasets to conduct cross-study analyses, preclinical modeling of adverse events, and the pros and cons of closed versus open data networks.

### Session IV: Collaboration across disciplines

#### Collaborative and integrated approach to Immuno-oncology biomarkers at PICI

Theresa LaVallee, PhD (Parker Institute for Cancer Immunotherapy (PICI), San Francisco, CA, USA) discussed PICI’s efforts to bring a cross-disciplinary approach to cancer immunotherapy guided by the field’s top scientists. For instance, one venture entails collaborative efforts to advance personalized cancer treatments through neoantigen discovery. Here, PICI has convened experts to identify optimal algorithms for prediction of neoantigens that will generate an immune response and has completed initial predictions regarding vaccine development for the treatment of patients with melanoma and NSCLC.

Increasing the field’s understanding of immune-related adverse events (irAEs) is also a major PICI goal. Researchers are beginning to understand that irAEs are not simply toxicities but are rather a pharmacodynamic activity of immune agents. Endocrinopathies, for example, are an example of a byproduct of an immune response that is related to the drug’s mechanism. To better understand and prevent irAEs, PICI is currently researching irAE mechanisms following checkpoint inhibition in cancer patients to identify at-risk patients early and determine if the irAE is similar to the natural presentation of the same pathological condition.

Additionally, PICI is invested in understanding the role of the microbiome in cancer. Emerging data reveal that a patient’s microbiome composition may correlate with response to anti-PD-1 therapy. PICI plans to launch a microbiome-cancer immunotherapy trial for patients with advanced melanoma in order to facilitate transition of microbiome analyses into the clinic [24].

#### Infectious disease and biomarkers I: Gates medical research institute

David Kaufman, MD, PhD (Bill & Melinda Gates Medical Research Institute, Cambridge, MA, USA) described progress in global health over the past couple of decades, including the near-elimination of polio, introduction of the meningitis vaccine into central Africa, cutting AIDS-related deaths in half, and significant drops in infant mortality in sub-Saharan Africa.

Many challenges remain in this area, however. To address these challenges, The Gates Foundation has launched the Bill & Melinda Gates Medical Research Institute—a ‘nonprofit biotech’ to drive the development of drugs and vaccines for tuberculosis, malaria and enteric disease. The Gates Medical Research Institute will develop small molecules, vaccines, biologics and biomarkers across pre-clinical and clinical settings with a focus on moving lead candidates through proof of concept clinical studies. The Gates Medical Research Institute plans to utilize many of the cutting-edge translational medicine and biomarker strategies currently being pioneered in the immuno-oncology field.

#### Infectious disease and biomarkers II: Viral biomarker discovery using CyTOF

Holden T. Maecker, PhD (Stanford University Medical Center, Stanford, CA, USA) discussed analyses of viral infection using CyTOF to elucidate specific T cell subsets correlating to an immune response against infectious disease. Markers indicating the presence of cytotoxic CD8+ T cells, multifunctional T cells, and central memory or effector memory T cells can all be analyzed using CyTOF, and without fluorescence spillover problems experienced in conventional flow cytometry. CyTOF disadvantages, however, include difficulties in analyzing very rare cell populations, and increased time for sample acquisition. Examples of recent efforts utilizing CyTOF include analyzing CMV-specific T cell profiles in lung transplant patients to predict whether they were at risk for viremia/rejection post-transplant, as well as predicting RSV vaccine response in elderly patients.

#### Biomarkers in autoimmunity

Zoe Quandt, MD (University of California San Francisco, San Francisco, CA, USA) discussed the use of biomarkers in combating irAEs due to autoimmune disease. Autoimmunity as a mechanism of pathogenesis is an alteration to immune function in terms of selection, T cell or B cell regulation, or an aberrant response to a particular antigen. Dr. Quandt’s research focuses on immune checkpoint inhibitor-induced diabetes mellitus (CPI-DM), which is largely observed in patients being treated with anti-PD1/PD-L1 therapy. Interestingly, in Dr. Quandt’s study, CPI-DM patients treated with checkpoint blockade experienced an autoantibody prevalence (generally thought to be non-pathogenic in DM) of 40% -- significantly lower than the autoantibody prevalence in conventional Type I Diabetes patients (95%). Further investigation revealed that patients treated with PD-1/PD-L1 inhibitors positive for autoantibodies developed earlier onset of CPI-DM, suggesting that these types of biomarker analyses may be able to help predict autoimmune-related irAEs [25].

#### Session IV panel discussion outcome

The panel weighed in on executing the Team TimIOS project, anticipated complications in acquiring and harmonizing clinical data, adopting new standards in clinical workflows, prioritizing access to data before perfecting the system, and deriving benefit from experts across disciplines to overcome current data sharing hurdles.

## Conclusions and next steps

Immunotherapy clinical trials are redefining how scientists and clinicians approach patient care. With the continued need for testing of novel agents and combination therapies, SITC’s Immune Biomarkers Committee reconvened to review and evaluate state of the art technologies, identify current hurdles, and to form working partnerships within the immunotherapy biomarkers arena. Throughout the workshop sessions, two major issues emerged - harmonization of data and novel biomarker discovery.

Harmonization and standardization of data generated from widely used assays and technologies is a major hurdle for the entire scientific community. From basic scientific discovery to clinical practice, there is a lack of universal standards on nomenclature, language, definitions, scoring, reporting, SOPs and global approvals, and a lack of comprehensive efforts to develop necessary standards. For instance, the lack of standardized diagnostic tests which measure biomarkers such as PD-L1 demonstrate the far reaching logistical and technical problems in improving therapeutic development and patient response - PD-L1 is currently measured by numerous assays with antibodies expressing various levels of positive expression. For this reason, institutional collaborations such as the described efforts between the CIMACs and CIDC share the objective of creating a harmonized, systematic approach to biomarker discovery, utilization and validation. Such networks share the ultimate goal of advancing correlative biological studies through the coordination of technological assays and protocols. Ultimately, harmonization of data will not only enable further investigation of scientific ideas and correlated discoveries but will lead to an eventual reduction in time and more efficient therapeutic development.

Furthermore, to overcome complex hurdles and encourage the development of personalized medicine, more sophisticated assays and systematic approaches are needed. Such cutting-edge technologies may be better equipped to address scientific and clinical issues, namely identification of novel biomarkers to better predict patient response to therapies. Thus, the second focus of discussion centered on biomarker discovery through emergent and transformative technologies. The workshop recognized many breakthrough technologies, including, but not limited to the MANAFEST assay which combines WES and TCR sequencing for determination of individual immune response, 3D tissue staining and microscopy to better explore the tumor microenvironment, MIBI for multiplex imaging using up to 100 antibodies, and high dimensional T cell profiling to identify antigen-specific T cells. Additionally, machine learning approaches such as the neoantigen fitness model and deep learning algorithms as applied to monitoring of biological immune markers in the 1000 Immunomes Project highlight evolving computational approaches. Such technologies are poised to test new hypotheses more rapidly and produce comprehensive informatics datasets in order to target and validate newly discovered biomarkers and drive therapeutic development.

Based on the data presented and the interactive discussion panels, several next steps were identified to address the bi-level discussion which emerged from the workshop. 1) Members of the existing SITC Biomarkers Task Force will identify SOPs and methodological publications to post to the SITC website as a resource. 2) Task Force members will work together to identify technologies and co-authors for an updated series of Biomarker Technology primers for JITC. 3) A new multispectral imaging task force will be created to share best practices in this technology platform to further its optimal utilization. 4) The images from SITC’s Immunoscore Validation Task Force will work to share these images for future analysis. 5) A new immune-oncology data sharing task force will also be convened by SITC.
